# Prostate specific membrane antigen (PSMA) expression in non-small cell lung cancer

**DOI:** 10.1371/journal.pone.0186280

**Published:** 2017-10-27

**Authors:** Lars Henning Schmidt, Birthe Heitkötter, Arik B. Schulze, Christoph Schliemann, Konrad Steinestel, Marcel Trautmann, Alessandro Marra, Ludger Hillejan, Michael Mohr, Georg Evers, Eva Wardelmann, Kambiz Rahbar, Dennis Görlich, Georg Lenz, Wolfgang E. Berdel, Wolfgang Hartmann, Rainer Wiewrodt, Sebastian Huss

**Affiliations:** 1 Department of Medicine A, Hematology, Oncology and Respiratory Medicine, University Hospital Muenster, Muenster, Germany; 2 Gerhard Domagk Institute of Pathology, University of Münster, Münster, Germany; 3 Institute of Pathology and Molecular Pathology, Bundeswehrkrankenhaus Ulm, Ulm, Germany; 4 Department of Thoracic Surgery, Klinikum Bremen-Ost, Bremen, Germany; 5 Department of Thoracic Surgery, Niels-Stensen-Kliniken, Ostercappeln, Germany; 6 Department of Nuclear Medicine, University Hospital Münster, Münster, Germany; 7 Institute of Biostatistics and Clinical Research, Westfaelische Wilhelms-Universitaet Muenster, Muenster, Germany; 8 Translational Oncology, University Hospital Muenster, Muenster, Germany; 9 Cluster of Excellence EXC 1003, Cells in Motion, Muenster, Germany; University of South Alabama Mitchell Cancer Institute, UNITED STATES

## Abstract

**Objectives:**

PSMA (prostate-specific membrane antigen) is overexpressed in prostate cancer cells and is reported to be a promising target for antibody-based radioligand therapy in patients with metastasized prostate cancer. Since PSMA expression is not restricted to prostate cancer, the underlying study investigates PSMA expression in non-small cell lung cancer (NSCLC).

**Material and methods:**

Immunohistochemistry was used to identify PSMA expression in n = 275 samples of NSCLC tissue specimens. By means of CD34 co-expression, the level of PSMA expression in tumor associated neovasculature was investigated. The impact of PSMA expression on clinicopathologic parameters and prognosis was evaluated.

**Results:**

PSMA tumor cell expression in NSCLC is as low as 6% and was predominantly found in squamous cell carcinoma (p = 0.002). Neovascular PSMA expression was found in 49% of NSCLC. High neovascular PSMA expression was associated with higher tumor grading (G3/G4) (p < 0.001). Neither for PSMA tumor cell expression, nor for PSMA neovascular cell expression prognostic effects were found for the investigated NSCLC cases.

**Conclusion:**

Here, we report on the expression of PSMA in NSCLC tissue samples. Against the background of a potential treatment with radiolabeled PSMA ligands, our data might serve for the future identification of patients who could benefit from this therapeutic option.

## Introduction

The fact that patients are often diagnosed with late-stage and/or metastatic disease contributes to the high mortality of lung cancer patients. Since lung cancer is the most frequent cause of cancer-related death, it is necessary to improve both our diagnostic and our therapeutic armamentarium to overcome its high lethality and its poor prognosis[1]. PSMA (prostate-specific membrane antigen) is a type II transmembrane glycoprotein with influence on the activity of folate hydrolase and neurocarboxypeptidase[2, 3]. Initially, upregulation of PSMA was found in prostate cancer cells. Consequently, PSMA-based imaging technologies for the detection of metastatic disease in advanced-stage prostate cancer patients were developed and PSMA-based radioligand therapy was introduced as a therapeutic regimen in metastasized prostate cancer[3–10].

In addition, PSMA is expressed in the endothelium of tumor-associated neovasculature of various solid malignancies (i.e. breast, lung and urothelial cancer), possibly due to tumor-associated angiogenic factors[11–15]. Functionally, PSMA might facilitate endothelial cell sprouting and invasion through its regulation of lytic proteases that have the ability to cleave the extracellular matrix[6, 16]. Moreover, the expression of PSMA in malignancy-associated neovasculature bears the possibility of specific antibody-based therapies.

The current study has its focus on the expression of PSMA in a comprehensive lung cancer study cohort of n = 275 patients. In the context of PSMA-targeted anti-tumor therapies, both neovascular and tumor cell PSMA expression were evaluated.

## Methods

### Study population

Lung cancer tissue samples and the corresponding clinical follow-up data of n = 275 curatively resected NSCLC patients (217 NSCLC patients from the Thoracic Departments in Ostercappeln, Germany; 58 NSCLC patients from the University Hospital Mainz) were collected and examined. Patients with stage IV, neoadjuvant treatment, R1 or R2 resection status, or with non-specified tumor histology (*e*.*g*. NSCLC not otherwise specified) were excluded from our analysis. All tissue samples are embedded in tissue microarrays. The original patient data set contains 304 patient samples. Due to loss of tissue sections from the arrays, immunohistochemical evaluation was not possible in 29 cases. Hence, 275 NSCLC patients were included for further evaluation. Approval of the study by the Ethical Committees in Mainz [837.188.99 (2133)] and Münster [Az 2016-445-f-S and Reg.Nr.:4XMüller1] were obtained. All data were fully anonymized before they were accessed and the local ethics committee waived the requirement for informed consent. Clinical TNM staging (including clinical examination, CT scans, sonography, endoscopy, MRI, bone scan) was based on IUCC/AJCC recommendations. In terms of definite tumor staging, pathological exploration was carried out post-surgically. Primary pulmonary lesions were pathologically classified based on the WHO 2004 guidelines; 121 specimens were classified as squamous cell carcinoma, 112 as adenocarcinoma and 42 as large cell carcinoma. Survival time was either computed from the date of histological diagnosis to death or censored at the date of last contact. Baseline information of the NSCLC population is shown in Table 1.

**Table 1:** Baseline characteristics of the NSCLC study population.

Parameter	n	% of non-missing values
**Age** (mean)	66.1 years	
**Sex**		
Female	66	24
Male	209	76
**Tumor cell PSMA expression**		
SI 0	258	94
SI 1	11	4
SI 2	5	2
SI 3	1	1
**Neovascular PSMA expression**		
SI 0	140	51
SI 1	92	34
SI 2	39	14
SI 3	4	2
**Performance status (WHO ECOG**		
ECOG 0	47	18
ECOG 1	202	77
ECOG 2	14	5
**Tumor Histology**		
Adenocarcinoma	112	41
Large cell carcinoma	42	15
Squamous cell carcinoma	121	44
**Tumor stage**		
UICC Tumor stage I	188	68
UICC Tumor stage II	60	22
UICC Tumor stage III	27	10
**Grading**		
G1	6	2
G2	94	35
G3	138	51
G4	31	12

### Immunohistochemistry

Immunohistochemistry (IHC) was performed on 4-μm-thick paraffin sections using the peroxidase-conjugated avidin-biotin method. Primary antibodies included a monoclonal mouse anti-PSMA antibody (clone 3E6, Ventana, Germany, 1:50 dilution), monoclonal anti-CD34 antibody (clone QBEnd10, Ventana, Germany, ready to use concentration of 0.8μg/ml) and anti-CD31 antibody (clone JC70, Cell Marque, United States, concentration of 0.61μg/ml). In brief, sections were deparaffinized in xylene and rehydrated through graded ethanol at room temperature. Incubation with the primary antibodies was performed for 30 minutes at room temperature. After washing, the paraffin sections were incubated with biotinylated secondary antibodies. Immunoreactions were visualized using a 3-amino-9-ethylcarbazole as a substrate (Ventana Optiview DAB IHC detection KIT, Germany). Prostate carcinoma tissue sections served as a positive control. Several anti-PSMA antibodies are currently available and the most commonly used subclones include 7E11 and 3E6. We decided to use clone 3E6 as it targets an extracellular epitope of PSMA. For imaging or therapeutic purposes only antibodies targeting extracellular domains can be potentially used as they have to be internalized by living cells.

### Assessment of PSMA expression

PSMA expression was evaluated by two pathologists (BH and KS). In lung cancer, tumor cells and associated neovascular endothelium were analyzed separately, and the identity of vascular structures was confirmed by CD34 coexpression, a marker for endothelial cells among others[17–19]. Any reactivity in either tumor cells or neoplastic vessels was considered positive. Staining intensity was scored semiquantitatively as negative (0), weak (1 = barely perceptible staining at high power (400x) magnification), moderate (2 = readily apparent at low power (40x) magnification) or strong (3). The fraction of PSMA positive cells was assessed as < 5% or ≥ 5%. In the case of heterogeneous staining, the predominant pattern was recorded. For further analysis, labeling indices were defined. A weak (1) or moderate (2) staining intensity in ≤ 5% of the neovasculature and a weak (1) staining intensity in > 5% of the neovasculature was allocated to the “low expression” group (PSMA labelling index = 1), whereas a moderate (2) staining intensity in > 5% of the neovasculature and a strong (3) staining intensity in ≤ or > 5% of the neovasculature were assigned to the “strong expression” group (PSMA labelling index = 2). This scoring system has been previously established in soft tissue tumors[20, 21].

### Statistics

SPSS (SPSS Statistics, Version 24.0 released 2016, IBM Corp., Armonk, United States) was used for all statistical analyses. The study population was described by standard descriptive statistical measures. For categorical variables, absolute and relative frequencies are reported. Continuous variables are described by mean, standard deviation, median and inter-quartile range (IQR). Survival time is defined from first diagnosis until death. Univariate overall survival analysis was performed using the Kaplan-Meier method and log rank tests. We considered potential prognostic factors that are tolerably complete (less than ten missing values, and with at least ten cases) to prevent statistical problems emerging from low sample size and extreme values. Patients with missing values in the cofactors were excluded from the analysis. All statistical tests were performed as exploratory analyses on a local significance level of 0.05. Since multiplicity adjustment was not carried out, no distinct overall significance level was ascertained. Hence, our findings may be used to set up new hypotheses.

## Results

### PSMA expression in NSCLC tissues

**Fig 1:**
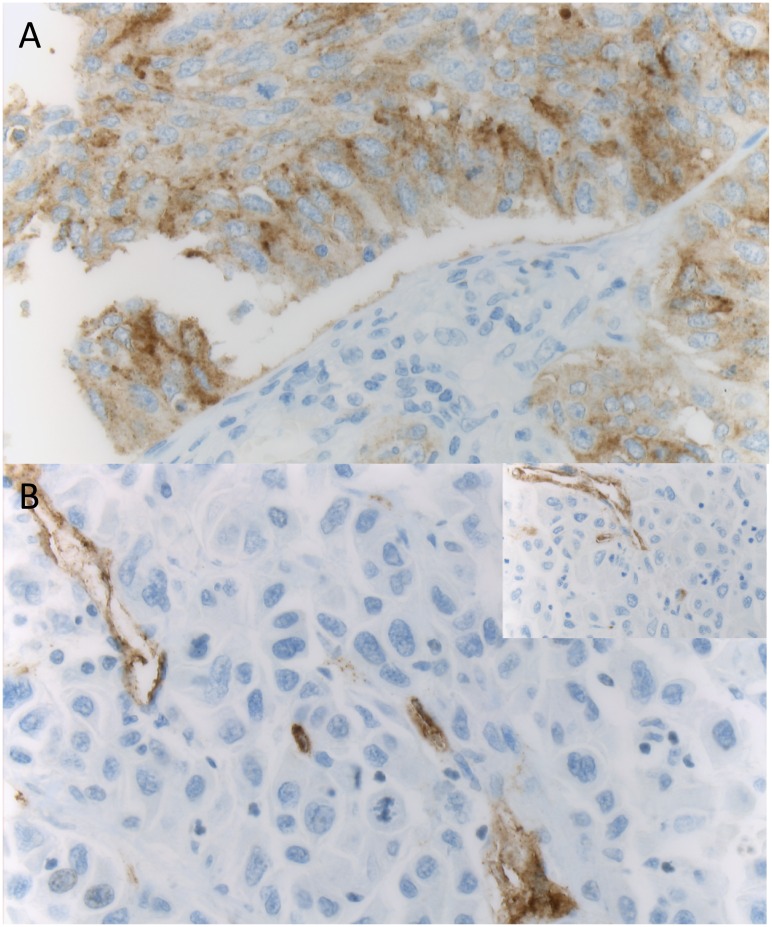
PSMA expression in non-small cell lung cancer (NSCLC). A (100x, anti-PSMA) displays strong PSMA expression in tumor cells. In B (100x, anti-PSMA), neovascular PSMA expression is shown. Neovasculature was identified by CD34 coexpression (insert, anti-CD34).

Baseline characteristics of n = 275 NSCLC patients with available immunohistochemical staining results are summarized in Table 1. A positive immunostaining (*i*.*e*. 2+/3+, according to the scoring system as described above) of tumor tissues with the monoclonal PSMA antibody was found in n = 17 cases (6%). Likewise, a positive immunostaining (*i*.*e*. 1+/2+/3+, according to the scoring system as described above) of neovascular PSMA expression was found in n = 135 cases (49%). In the 14 cases with PSMA-positive squamous lung cancer cells the tumor associated neovasculature was expressing PSMA in 8 cases. A representative example for PSMA expression is demonstrated in Fig 1.

### Clinicopathological correlations

With regard to clinicopathological parameters, positive correlations for tumor cell PSMA expression (i.e. 2+/3+) were found for tumor histology. In total n = 17 patients displayed tumor cell PSMA expression. Among these patients, n = 14 patients suffered from squamous cell carcinoma (82%; p = 0.002, two-sided Fisher’s exact test). In contrast, tumor cell PSMA expression in adenocarcinoma and in large cell carcinoma was low (Table 2). With regard to tumor histology, neovascular PSMA expression also displayed a positive correlation. Here n = 69 patients with squamous cell carcinoma (51%) expressed PSMA in tumor-associated neovasculature, whereas n = 88 patients with non-squamous cell carcinoma (63%) were negative for neovascular PSMA expression (p = 0.021, two-sided Fisher’s exact test). Furthermore, there was a positive correlation between high histological grade and PSMA expression, with PSMA-positive neovasculature found more frequently in high-grade or undifferentiated tumors. Of those, n = 99 tumor samples (75%) displayed positive neovascular PSMA expression (p < 0.001, two-sided Fisher’s exact test). The relationship of of neovascular PSMA staining and grading depending on the histological subtype is shown in supplementary S1 Table. Neither for tumor stage, nor for patient age relevant associations could be found (two-sided Fisher’s exact test for all: p > 0.05; Table 2). Of interest, of the n = 17 patients with positive tumor cell PSMA expression n = 13 patients were male (76%; p > 0.05, two-sided Fisher’s exact test) and n = 112 patients with positive neovascular PSMA expression (83%) were male (p = 0.011, two-sided Fisher’s exact test; Table 2).

**Table 2:** Correlations of clinicopathological variables with PSMA in NSCLC patients.

Parameter	Tumor cell PSMA expression			Neovascular PSMA expression		
	SI 0/1	SI 2/3	p-value*	SI 0/1	SI 2/3	p-value*
**Age**			0.793			0.899
<70 years	168	10		90	88	
≥70 years	89	6		49	46	
**Sex**			1.000			0.011
Male	196	13		97	112	
Female	62	4		43	23	
**Performance status**			1.000			0.108
ECOG 0	45	2		29	18	
ECOG 1–3	203	13		104	112	
**Tumor Histology**			0.002			0.021
Non squamous cell carcinoma	151	3		88	66	
Squamous cell carcinoma	107	14		52	69	
			0.011			0.001
Non adenocarcinoma	148	15		69	94	
Adenocarcinoma	110	2		71	41	
			0.484			0.180
Non large cell carcinoma	217	16		123	110	
Large cell carcinoma	41	1		17	25	
**Tumor stage**			0.284			0.605
UICC Tumor stage I	174	14		98	90	
UICC Tumor stage II-III	84	3		42	45	
**Grading**			1.000			<0.001
G1/G2	94	6		67	30	
G3/G4	159	10		70	99	

*p-value according to two-sided Fisher’s exact test

### Univariate prognostic effects

**Fig 2:**
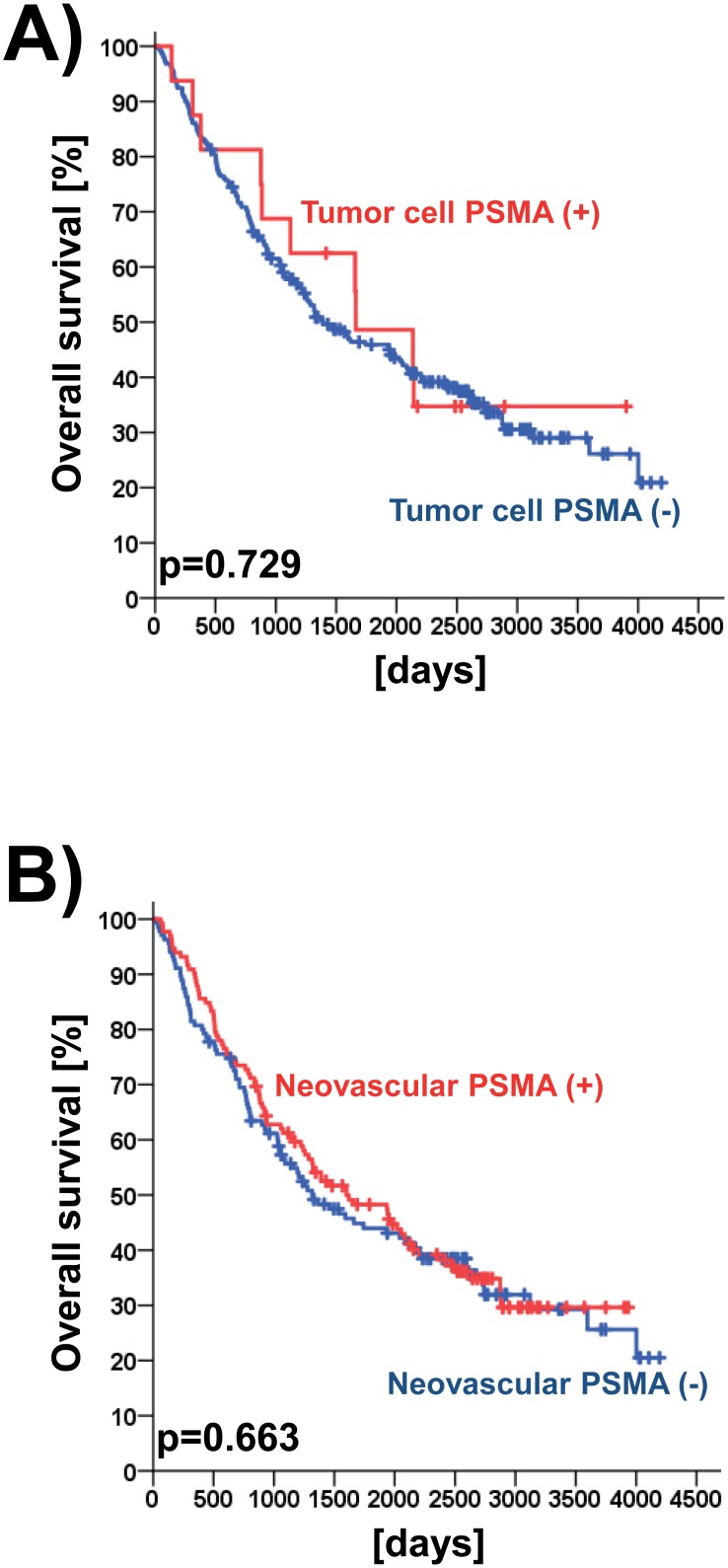
Prognostic impact of PSMA expression in non-small cell lung cancer (NSCLC). Univariate prognostic models are shown for the immunohistochemical staining results (Fig 2A). Likewise prognostic models are given for neovascular PSMA expression of all patients as detected by immunohistochemistry (Fig 2B).

In the entire NSCLC study collective, neither tumor cell PSMA expression nor neovascular PSMA expression had a prognostic effect (both analyses p > 0.05; log rank test). Besides, no subgroup depending prognostic (i.e. tumor histology, grading and tumor stage) effects were found (p > 0.05; log rank test; Table 3). To visualize prognostic effects, Kaplan-Meier curves were generated (Fig 2A and 2B).

**Table 3:** Univariate log rank test results for the association of PSMA with overall survival for defined subgroups. The investigated study collective consisted of n = 275 tissue microarray-embedded NSCLC specimens (immunohistochemical analysis).

Parameter	Tumor cell PSMA expression	Neovascular PSMA expression
	SI 0/1 *vs*. SI 2/3	SI 0/1 *vs*. SI 2/3
**All investigated patients**	0.729	0.663
**Tumor Histology**		
Non squamous cell carcinoma	0.149	0.767
Squamous cell carcinoma	0.986	0.169
**Tumor stage**		
UICC Tumor stage I	0.763	0.201
UICC Tumor stage II-III	0.607	0.341
**Grading**		
G1/G2	0.207	0.347
G3/G4	0.427	0.065

## Discussion

To overcome the poor prognosis of lung cancer patients, it is crucial to both improve the prognostic stratification of patients at the diagnostic level and to expand our therapeutic armamentarium. In this context, molecular targeted therapies on the basis of tumor-associated antigens are a promising approach.

Among suitable candidates for targeted therapy concepts, the type II transmembrane protein PSMA might be a promising target antigen in lung cancer[22]. Two facts contribute to this strategy: (1) Upon ligand binding and subsequent endocytosis, PSMA is transported directly into the cells[23]. (2) Whereas strong PSMA expression is found in prostatic cancer cells (including advanced-stage prostate carcinomas)[24], normal tissue displays rather low expression levels[22]. With regard to these facts, enhanced uptake levels and retention in the tumor favor the local enrichment of antitumoral compounds. Hence, PSMA might serve as an ideal candidate for targeted radiopharmaceutical therapies[22]. Besides prostate carcinoma, PSMA expression has been detected in other malignancies[11–15]. Functionally, PSMA has been shown to be involved in angiogenesis and endothelial cell sprouting[6, 16], but its exact role in tumor-associated angiogenesis remain to be further clarified[25].

To identify the prognostic relevance of PSMA, we performed a systematic analysis of a comprehensive NSCLC study cohort (n = 275) with focus on the PSMA expression in both neovascular and tumor cells. In our study collective, PSMA tumor cell expression was found in n = 17 cases (6%).

To our knowledge, there is only one additional study that reports on PSMA expression in lung cancer. Wang *et al*. describe high PSMA expression rates both for tumor neovasculature endothelial cells (85%) and for tumor cells (54%) in n = 150 NSCLC patients. Besides, PSMA-positive endothelial cells were found more often in early-stage NSCLC in contrast to advanced NSCLC tissues[12].

Similar to the published results, we detected strong neovascular PSMA expression levels in 49% of the investigated cases. However, the rate of NSCLCs with PSMA expression on tumor cells was much lower in our cohort (6%). The observed differences for PSMA expression on tumor cells might be due to a difference in the monoclonal antibodies that had been used in the different studies, heterogeneity of the investigated NSCLC study cohort and/ or differences in interpretation. In our study we used an anti-PSMA antibody (subclone 3E6) targeting the extracellular region of PSMA. In contrast to other subclones, it might be better used to predict the likely hood of success of PSMA imaging and therapy.

With regard to PMSA expression, it is difficult not to misinterpret antibody reaction products in macrophages as positive tumor cells. Of interest, PSMA tumor cell expression was detected more often in squamous cell carcinomas than in non-squamous NSCLC samples.

In our cohort, neovascular PSMA expression was detected in 49% of the investigated tumors. Overall, neovascular PSMA expression was found to be associated with higher histologic grade (p<0.001). Recently, we reported on PSMA expression patterns in soft tissue tumors[20]. In the latter study, higher histologic grade (e.g., poor tumor differentiation) had also been associated with higher neovascular PSMA expression. However, the biological relevance of this observation to be further clarified. According to one hypothesis, it might be due to intratumoral hypoxia as a result of rapid tumor growth. Since PSMA facilitates endothelial cell invasion during angiogenic sprouting, PSMA upregulation might enhance tumor vascularization in this setting[6, 16]. As a result, targeting PSMA-expressing neovessels might represent a promising therapeutic option in rapidly growing solid tumors.

For patients with metastatic prostate cancer, the PSMA-targeted radionuclide therapy has been shown to be a therapeutic and diagnostic option[4, 26, 27]. In the light of our findings, patients with PSMA expressing NSCLC tumors might benefit from PSMA-targeted radionuclide therapies. However, given the low frequency of PSMA expressing tumors (6% in our cohort, predominantly squamous cell carcinomas), prospective studies should focus on the immunohistochemical pre-evaluation in order to identify candidates with possible benefit from these targeted therapeutic approaches. Moreover, our findings of PSMA expression in tumor-associated neovasculature of 49% of the investigated cases might point towards a possible anti-angiogenic effects of PSMA-targeted radionuclide therapy in these patients.

In line with this theory, PSMA-targeted therapies in different non-prostate cancers have been recently published. Both, radionuclide based PSMA antibodies and PSMA-targeted docetaxel-containing nanoparticle were used and preliminary evaluated[28, 29].

In conclusion, our study reports the expression of PSMA in a subset of NSCLCs, especially in tumor-associated neovasculature. Expression does not seem to “drive” disease, but the presence of PSMA in these selected cases might represent a potential therapeutic target. With focus on the treatment with radiolabeled PSMA ligands, our data might serve for the identification of patients who might benefit from these novel and promising therapeutic approaches.

## Supporting information

S1 Table Correlation of clinicopathological variables with PSMA expression in NSCLC patients depending on tumor histology and grading. (DOCX)

S2 Table Full data sample. (XLSX)
