# Supplementary material for: Prostate specific membrane antigen (PSMA) expression in non-small cell lung cancer
Source: PLoS One. 2017 Oct 27;12(10):e0186280. doi: 10.1371/journal.pone.0186280 (PMC5659610; doi:10.1371/journal.pone.0186280)
Supplement: S1 Table — (DOCX) [file pone.0186280.s001.docx]

**S1 Table. Correlation of clinicopathological variables with PSMA expression in NSCLC patients depending on tumor histology and grading.**

**A)**

|  | **Tumor cell PSMA expression** | | |
| --- | --- | --- | --- |
| **Tumor Histology** | **SI 0/1** | **SI 2/3** | **p-value*** |
| Squamous cell carcinoma |  |  | 0.550 |
| - G1/G2 | 30 | 5 |  |
| - G3/G4 | 75 | 9 |  |
| Adenocarcinoma |  |  | 1.000 |
| - G1/G2 | 62 | 1 |  |
| - G3/G4 | 48 | 1 |  |
| Large cell carcinoma |  |  | n.e. |
| - G1/G2 | 2 | 0 |  |
| - G3/G4 | 36 | 0 |  |

**B)**

|  | **Neovascular PSMA expression** | | |
| --- | --- | --- | --- |
| **Tumor Histology** | **SI 0/1** | **SI 2/3** | **p-value*** |
| Squamous cell carcinoma |  |  | 0.103 |
| - G1/G2 | 19 | 16 |  |
| - G3/G4 | 31 | 53 |  |
| Adenocarcinoma |  |  | 0.006 |
| - G1/G2 | 47 | 16 |  |
| - G3/G4 | 24 | 25 |  |
| Large cell carcinoma |  |  | 1.000 |
| - G1/G2 | 1 | 1 |  |
| - G3/G4 | 15 | 21 |  |

*p-value according to two-sided Fisher’s exact test

n.e. = not evaluable
